# Which aspects of health care are most valued by people living with HIV in high-income countries? A systematic review

**DOI:** 10.1186/s12913-016-1914-4

**Published:** 2016-11-30

**Authors:** V. Cooper, J. Clatworthy, E. Youssef, C. Llewellyn, A. Miners, M. Lagarde, M. Sachikonye, N. Perry, E. Nixon, A. Pollard, C. Sabin, C. Foreman, M. Fisher

**Affiliations:** 1 Elton John Centre, Sussex House, Brighton and Sussex University Hospitals NHS Trust, 1 Abbey Road, Brighton, BN2 1ES UK; 2 Division of Public Health & Primary Care, Brighton & Sussex Medical School, Room 317 Mayfield House, Falmer, Brighton, BN1 9PH UK; 3 Health Services Research Unit, London School of Hygiene and Tropical Medicine, London, WC1E 7HT UK; 4 Health Services Research Unit, Global Health and Development, London School of Hygiene and Tropical Medicine, London, WC1E 9SY UK; 5 UK Community Advisory Board Country United Kingdom (England), c/o HIV i-Base, 4th Floor, 57 Great Suffolk Street, London, SE1 0BB UK; 6 UCL Medical School, Infection and Population Health, University College London, Royal Free Campus, Rowland Hill Street, London, NW3 2PF UK; 7 NHS England, Southside - Mezzanine Floor, 105 Victoria Street, London, SW1E 6QT UK

**Keywords:** HIV, Ageing, Comorbidities, Healthcare services, Patient preferences, Systematic review

## Abstract

**Background:**

Increasing numbers of people with HIV are living into older age and experiencing comorbidities. The development of new models of care to meet the needs of this population is now a priority. It is important that the views and preferences of patients inform the development of services in order to maintain high levels of patient satisfaction and engagement. The aim of this systematic review was to determine which aspects of healthcare are particularly valued by people living with HIV.

**Methods:**

We searched electronic databases and reference lists of relevant articles. The search strategy was developed to identify articles reporting on HIV positive patients’ perceptions, evaluations or experiences of healthcare services and factors associated with satisfaction with care. Peer-reviewed papers and conference abstracts were included if the study reported on aspects of health care that were valued by people living with HIV, data were collected during the era of combination therapy (from 1996 onwards), and the paper was published in English. A thematic approach to data synthesis was used.

**Results:**

Twenty-three studies met the inclusion criteria. Studies used both qualitative and quantitative methods. Six studies specifically reported on relative importance to patients of different aspects of care. The valued aspects of care identified were grouped into seven themes. These highlighted the importance to patients of: a good health care professional-patient relationship, HIV specialist knowledge, continuity of care, ease of access to services, access to high quality information and support, effective co-ordination between HIV specialists and other healthcare professionals, and involvement in decisions about treatment and care. We were unable to determine the relative importance to patients of different aspects of care because of methodological differences between the studies.

**Conclusions:**

This review identified several attributes of healthcare that are valued by people living with HIV, many of which would be relevant to any future reconfiguration of services to meet the needs of an ageing population. Further research is required to determine the relative importance to patients of different aspects of care.

## Background

Over the past two decades, increasingly effective antiretroviral therapy (ART) has transformed HIV from a life-threatening illness with uncertain outcomes to a manageable long-term condition [1].

With timely diagnosis and appropriate treatment, the life expectancy of people living with HIV is now similar to that of HIV-negative individuals [2]. In line with these developments, there has been a change in health service use among people living with HIV, with a decrease in service use associated with opportunistic infections and an increase in use associated with comorbid illness [3, 4]. Indeed, as people are living longer with HIV, a growing number of people are now living with multiple chronic conditions [5–7].

In light of these changes, traditional models of HIV care, that have been predominately provided by HIV specialists, may no longer meet patients’ needs. The development of new models of care for people living with HIV is now a recognized priority [8]. Current guidelines suggest that it is important to link with non-HIV specialists to provide the best treatment for many non-HIV related conditions [9] - such as hypertension and depression -although the majority of primary care physicians think that patients would prefer their care to be managed by HIV specialists [10].

It is vital that the views and preferences of people living with HIV inform service development [11]. People living with HIV have typically reported high levels of satisfaction with specialist HIV services [12]. This is important because satisfaction has been associated with retention in HIV care, higher adherence to ART and improved clinical outcome (viral load suppression) [13]. Through understanding the aspects of care that are of particular importance to people living with HIV, it may be possible to develop new models of care that maintain these high levels of satisfaction and engagement with care.

The aim of this systematic review was to investigate which aspects of healthcare are particularly valued by people living with HIV.

## Methods

### Information sources and search strategy

This mixed-methods review was based on a systematic search of six online databases including Medline, PsycINFO, CINAHL, Cochrane, Embase, Web of Science using the terms listed in Table 1. The search strategy was developed to identify quantitative and qualitative articles reporting on HIV positive patients’ perceptions, evaluations or experiences of, or satisfaction with, healthcare services. The search covered the dates 1996 (the year in which combination antiretroviral therapy was introduced in the UK) to August 2015. Articles published before 1996 were not included because we were interested in perceptions of care in the era of effective HIV treatment. No ethical approval was required as this is a systematic review.

**Table 1:** Search Terms

HIV	AND	Satisf*	NEAR/5	Care
		Aspect*		Healthcare
		View*		service*
		Perception*		provider*
		Perceive*		
		Attitude*		
		Experience*		
		Belief*		
		Evaluat*		
		Value*		
		Prefer*		

*denotes truncation used in the search strategy e.g. satisf* finds satisfy, satisfied, satisfaction, satisfactory etc

### Eligibility and study selection

The titles and abstracts of retrieved papers were screened in order to exclude those that clearly did not meet the selection criteria, listed in Table 2. This process was conducted by two researchers (JC and VC), with 20% overlap in order to check reliability. There was 99% agreement between the two reviewers - disagreement was resolved through discussion. Full text copies were obtained when the articles appeared potentially relevant based on the abstract review. All papers were reviewed against the selection criteria by the same two researchers in collaboration, and those that met the criteria were retained for data extraction.

**Table 2:** Selection criteria

Inclusion criteria	A primary aim of the paper/element of the results was to explore which aspects of health care are valued by people living with HIV
	Data collected during the era of combination antiretroviral therapy (ART) (from 1996 onwards)
	Quantitative or qualitative methodology
	Written in English
	Published in a peer-reviewed journal or conference abstract
Exclusion criteria	Based on data collected prior to the introduction of combination ART (1996)
	Did not contain any primary data (e.g. review articles, editorials)
	Conference abstract without extractable data
	Research conducted outside of UK/Europe/USA/Canada/Australia/New Zealand
	Patients were children/adolescents
	Focus on dental care
	Focus on HIV care during pregnancy
	Focus on HIV testing services
	Focus on end of life care
	Focus on barriers to service entry
	Focus on inpatient services.

### Data extraction

After reading the papers, the two reviewers (JC and VC) agreed on a set of seven themes that encompassed the various aspects of care addressed (relationship with health care provider, expertise of health care provider, practical considerations, provision of information and support, coordination between services, factors relating to confidentiality/stigma and involvement in treatment decisions). To facilitate data synthesis, extracted data were organized in an excel spreadsheet according to these themes. The following data were also collected: study authors, year of publication, country, study aim; service type (e.g. outpatient HIV service, primary care); whether or not there was a specific sample characteristic (e.g. asylum seekers or intravenous drug users); sample size; gender and ethnicity.

### Quality assessment

Two researchers (VC and EY) independently rated the quality of individual studies, using the Mixed Methods Assessment Tool [MMAT] [14], which has been designed for mixed studies reviews. For each type of study (qualitative and quantitative descriptive studies), four items were used to assess quality (Table 3). For mixed methods studies, both quantitative and qualitative methods were assessed. For each of 4 items, response categories were ‘yes’, ‘no’ or ‘can’t tell. Each study received a score ranging from 25% (*) (1 criterion met) to 100% (****) (all criteria met). For mixed method studies, the overall quality score was the lowest score of the quantitative and qualitative components. No study was excluded on the basis of the quality assessment because we were interested in collating all aspects of care that have been identified as being important to people living with HIV.

**Table 3:** Quality assessment using the Mixed Methods Appraisal Tool

First author/year	Type of study	Screening questions		Qualitative				Quantitative descriptive			
		Are there clear qualitative or quantitative research questions, or a clear mixed methods research question?	Do the data collected address the research question?	1.1 Are the sources of qualitative data relevant to address the research question?	1.2 Is the process for analysing qualitative data relevant to address research question?	1.3 Is appropriate consideration given to how findings relate to the context in which data were collected?	1.4 Is appropriate consideration given to how findings relate to researchers’ influence through interaction with participants?	4.1 Is the sampling strategy relevant to address the research question?	4.2 Is the sample representative of the population under study?	4.3 Are measurements appropriate (clear origin, or validity known, or standard instrument)?	4.4 Is there an acceptable response rate (60% or above?)
Allan (2005) [20]	Qualitative**	Yes	Yes	Can’t tell	Yes	Yes	Can’t tell	-	-	-	-
Baker (2014) [18]	Quantitative**	Yes	Yes	-	-	-	-	Yes	Can’t tell	Yes	Can’t tell
Bennett (2014) [27]	Qualitative**	Yes	Yes	Yes	Yes	Can’t tell	Can’t tell	-	-	-	-
Bodenlos (2004) [34]	Quantitative****	Yes	Yes	-	-	-	-	Yes	Yes	Yes	Yes
Dang (2012) [12]	Quantitative****	Yes	Yes	-	-	-	-	Yes	Yes	Yes	Yes
Davis-Michaud (2004) [22]	Mixed*	Yes	Yes	No	Yes	Can’t tell	Can’t tell	Can’t tell	No	Yes	Can’t tell
Dawson-Rose (2005) [21]	Qualitative***	Yes	Yes	Yes	Yes	Yes	Can’t tell	-	-	-	-
Emlet (2002) [30]	Quantitative***	Yes	Yes	-	-	-	-	Yes	Yes	Yes	No
Harrison (2009) [31]	Quantitative*	Yes	Yes	-	-	-	-	Can’t tell	Can’t tell	Yes	Can’t tell
Hekkink (2003) [23]	Mixed***	Yes	Yes	Yes	Yes	Can’t tell	Can’t tell	Yes	Yes	Yes	No
Hekkink (2005) [32]	Quantitative****	Yes	Yes	-	-	-	-	Yes	Yes	Yes	Yes
Hope (2001) [33]	Quantitative***	Yes	Yes	-	-	-	-	Yes	Can’t tell	Yes	Yes
Laschinger (2005) [24]	Qualitative**	Yes	Yes	Can’t tell	Yes	Can’t tell	Can’t tell	-	-	-	-
Mallinson (2007) [25]	Qualitative****	Yes	Yes	Yes	Yes	Yes	Can’t tell	-	-	-	-
McCoy (2005) [26]	Qualitative****	Yes	Yes	Yes	Yes	Yes	Yes	-	-	-	-
Moore (2010) [15]	Mixed**	Yes	Yes	Yes	Yes	Can’t tell	Can’t tell	Can’t tell	No	Yes	Yes
Ndirangu (2009) [16]	Qualitative***	Yes	Yes	Yes	Yes	Can’t tell	Can’t tell	-	-	-	-
Pollard (2015) [29]	Qualitative***	Yes	Yes	Yes	Yes	Yes	Can’t tell	-	-	-	-
Sullivan (2000) [35]	Quantitative****	Yes	Yes	-	-	-		Yes	Yes	Yes	Yes
Tsasis (2010)	Quantitative**	Yes	Yes	-	-	-	-	Yes	Can’t tell	Can’t tell	Yes
Vyavaharkar (2008) [17]	Qualitative***	Yes	Yes	Yes	Yes	Yes	Can’t tell	-	-	-	-
Williams (2011) [28]	Qualitative*	Yes	Yes	Yes	Can’t tell	Can’t tell	Can’t tell	-	-	-	-
Zablosta (2009)	Quantitative**	Yes	Yes	-	-	-	-	Yes	Can’t tell	Yes	No

For qualitative and quantitative studies:* = one criterion met;** = 2 criteria met; *** = 3 criteria met; **** = 4 criteria met; For mixed methods studies the quality score is the lowest score of the study components:* = one criterion met for either qualitative or quantitative components;** = 2 criteria met for either qualitative or quantitative components;*** = 3 criteria met for either qualitative or quantitative components**** = 4 criteria met for both qualitative or quantitative components

### Assessment of risk of bias across studies

There was a risk of bias across studies, including publication of positive results and selective reporting of data within studies. Since the data reported were descriptive, we did not use any statistical analyses such as sensitivity analyses or subgroup analyses to control for bias.

## Results

### Study selection

**Fig 1:**
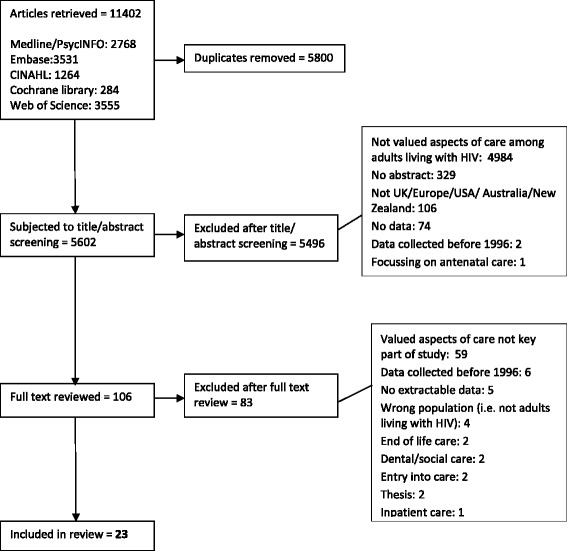
Study selection: Number of articles retrieved and excluded at each stage

The numbers of articles retrieved from the search, screened, assessed for eligibility and included in the review, with reasons for exclusion at each stage, are shown in Fig. 1.

### Study characteristics

Table 4 provides an overview of the 23 studies that met the inclusion criteria. Most explored valued aspects of care among a general sample of people living with HIV but some focused on the views of specific groups, such as those from Black African communities [15–18], men who have sex with men (MSM) [19], asylum seekers [20] and intravenous drug users [21].

**Table 4:** Overview of the studies reviewed

Study	Country	Aim relevant to this review	Methods	Service type	HIV+ Sample	N (HIV+)	Mean Age (Years)	Gender (% male)	Ethnicity
Allan & Clarke (2005)	UK	To determine whether existing HIV services in Leeds meet the needs of HIV-positive asylum seekers.	Qualitative: Interviews	GUM service	Asylum seekers	14	Not stated	Not stated	Not stated
Baker et al. (2014)	USA	To analyse satisfaction with health care among African American women living with HIV/AIDS	Quantitative: Questionnaire	HIV outpatient clinic	African American women	157	40 (SD 9)	0	100% African American
Bennett et al. (2014)	UK	To explore the experience and needs of people living with HIV who are accessing healthcare services.	Qualitative: Focus groups	Not stated	General	16	Not stated	44	Not stated
Bodenlos et al. (2004)	USA	To develop and validate an instrument to measure patient attitudes toward Health Care Professionals in the HIV population.	Quantitative: Questionnaire	Outpatient clinic	General	129	38 (median) (Range 18–61)	57	83% African American 16% Caucasian 1% Hispanic
Dang et al. (2012)	USA	To determine components which contribute to patients’ satisfaction with HIV care and the relative importance of each component.	Quantitative: Questionnaire	HIV outpatient service	General	489	48 (SD 11)	71	61% Non Hispanic Black 15% Non Hispanic White 21% Hispanic 3% Other
Davis-Michaud et al. (2004)	USA	To explore patient preferences regarding HIV care.	Qualitative and quantitative: Focus groups and ranking exercise	Not specified	General	29	41 (Range 26–60)	69	25% African American 7% Latino 62% Caucasian 3% Asian 3% Native American
Dawson-Rose et al. (2005)	USA	To identify barriers and facilitators to care among HIV positive injection drug users.	Qualitative: Interviews	Not specified	Injection drug users	161	35 (SD 7)	50	62 % African American 13% Latino 21% Caucasian 4% Mixed/Other
Emlet & Berghuis (2002)	USA	To explore service use differences between younger and older persons with HIV/AIDS?	Quantitative: Questionnaire	Not specified	General (Divided into groups based on age)	287	Younger group 34 (SD 3.9) Older group 54 (SD 4.5)	Younger group 86 Older group 94	Younger group 70% White 29% Non-white Older group 78% White 21% Non-white
Harrison et al. (2009)	UK	To conduct a patient survey to help design a new HIV/Sexual Health service.	Quantitative: Questionnaire	HIV/sexual health outpatient clinic	General	59	Not stated	Not stated	38% African
Hekkink et al. (2003)	Netherlands	To develop and validate a questionnaire to measure the quality of HIV care from the patient’s perspective.	Qualitative and quantitative: Focus groups and questionnaire	Not specified	General	Focus groups 15 Questionnaire 44	Focus groups 49 (Range 30–62) Questionnaire 43 (SD 7.6)	Focus groups 80 Questionnaire 84	Not stated
Hekkink et al. (2005)	Netherlands	To compare patients’ perceptions of the quality of HIV care received from nursing consultants, HIV specialists and GPs.	Quantitative: Questionnaire	GP and specialist HIV care	General	153	44 (SD 7.4)	90	Not stated
Hope et al. (2001)	UK	To collect data to inform the improvement of HIV/GUM services in West London.	Quantitative: Questionnaire	HIV/GUM outpatient clinic	General	202	16 % ≤30 yrs 84 % > 30 yrs	88	82% White 8% Black 6% Mixed race 3% Asian
Laschinger et al. (2005)	Canada	To describe and compare perceptions of HIV care from the perspectives of patients and health care professionals.	Qualitative: Focus groups	HIV/mixed outpatient clinics	General	Not stated	Not stated	Not stated	Not stated
Mallinson et al. (2007)	USA	To discover what specific provider behaviours influence engagement in HIV care from the client’s perspective.	Qualitative: Interviews	Community services/clinics	General	76	39 (Range 19–58)	51	51% African American 19% Hispanic 13% Mixed race 12% White/Caucasian 4% Native American 1% Asian
McCoy (2005)	Canada	To explore HIV patients' perceptions of 'good doctoring'.	Qualitative: Interviews and focus groups	Community services/clinics	General	79	Early 20s to late 50s	72	Not stated
Moore et al. (2010)	USA	To assess the value of the QUOTE-HIV questionnaire to identify African American patients’ perceptions of HIV care and further explore health care disparities in the HIV-positive African American population.	Qualitative and quantitative: Questionnaire and focus groups	Mixed	African-Americans	Questionnaire 55 Interviews 16	Range 20-59	69	100% African American
Pollard et al. (2015)	UK	To examine patients’ preferences for the future delivery of services	Qualitative: Focus groups	HIV outpatient clinic	General	74	Not stated	61	41% White British 4% White other 41% Black African 7% Other Black 3% Mixed race
Ndirangu & Evans (2009)	UK	To explore migrant African women's experiences of coping with HIV and their views about the HIV services.	Qualitative: Interviews	Hospital clinic/drop in centre	African women living in the UK	8	Range 30s-50	0	62% Zimbabwean 13% Congolese 25% Malawian
Sullivan et al. (2000)	USA	To explore the extent to which various aspects of the doctor-patient relationship were associated with overall satisfaction with the doctor.	Quantitative: Two satisfaction questions	Outpatient clinic	General	146	37 (SD 7.9)	75	49% Black 21% Hispanic 30% White
Tsasis et al. (2000)	Canada	To explore factors associated with satisfaction with HIV care.	Quantitative: Questionnaire	Outpatient clinic	General	193	Majority aged 30–49 years	91	Not stated
Vyavaharkar et al. (2008)	USA	To explore the perceptions of the availability, accessibility, and quality of HIV health care and social services of African American women residing in rural South Carolina.	Qualitative: Focus groups	Not stated	African-American women	22	44 (SD 9.2)	0	100% African American
Williams et al. (2011)	USA	To determine the barriers to and facilitators of consistently attending HIV medical care visits among a group of PLWH who had successfully negotiated enrolling in HIV care.	Qualitative: Focus groups	Public infectious disease clinic	General	25	40 (Range 24–54)	60	84% African American
Zablotska et al. (2009)	Australia	To explore service needs of gay men living with HIV and any barriers to accessing them.	Quantitative: Questionnaire	Mixed (GP/outpatient services/sexual health clinics)	Men who have sex with men	270	46 (median) (Range 26–72)	100	Not stated

GP = General practitioner; GUM = Genitourinary Medicine

Thirteen of the studies used interviews and/or focus groups to gather qualitative data about aspects of care that are particularly valued [15–17, 20–29]. Thirteen quantitative descriptive studies used questionnaires to collect data [13, 15, 18, 19, 22, 23, 30–36]. Three studies used a mixed methods design [15, 22, 23]. Six studies asked patients to rate the importance of various aspects of care [13, 15, 22, 23, 32, 33]. One study used a card sorting exercise to determine the relative importance of different aspects of care [22].

### Quality assessment

The quality of studies ranged from 25% to 100% (Table 3). Of the 23 studies included, 13 (57%) met three or four of the four quality criteria and were deemed to be of good quality. Seventeen (74%) of studies lacked detail on one or more aspects of the methodology used. For qualitative studies there was little information on the interaction between the researcher and participants [15–17, 20–25, 27–29] and how findings related to the context in which the data were collected [15, 22–24, 27, 28].

For quantitative descriptive studies, response rates were often lower than 60% or not reported [18, 19, 22, 23, 30, 31], the sample strategy method was not described [15, 22, 31], and it was not always clear whether the sample was representative of the population under study [15, 18, 19, 22, 31, 33, 36].

### Data synthesis

Aspects of care identified in the studies were grouped into seven themes: relationship with health care provider, expertise of health care provider, practical considerations, provision of information and support, coordination between services, factors relating to confidentiality/stigma and involvement in treatment decisions. These themes are described in more detail below.

### 1. Relationship with health care provider

Of the 23 studies included in the review, 19 (83%) cited valued aspects of the relationship between patients and health care providers (HCPs). Twelve (63%) of these studies were rated as being of good quality.

Interpersonal aspects of care rated important by people living with HIV in quantitative studies included professionalism [35], taking patients seriously [23, 32], providing emotional support [34], taking an interest in personal relationships, empathy and enabling patients to feel comfortable discussing personal issues [35].

Qualitative studies shed further light on the aspects of the HCP-patient relationship that were valued by people with HIV. Patients emphasised the importance of building a good relationship with their HIV doctor [27], with trust being a key feature of the relationship [20, 25]. Continuity was important – patients preferred to see the same HCP at each appointment to avoid having to repeat their story to someone who did not fully understand their needs [24, 27].

Important personal qualities of HCPs included being caring, compassionate, approachable, friendly, familiar, respectful, understanding, supportive and having a positive attitude [15, 20, 21, 24, 26–28]. Valued behaviours included making eye contact, smiling, showing concern, spending time talking to the patient and speaking kindly to the patient [15, 16, 24, 26]. Patients highlighted the importance of being treated as an individual [24–26]. One paper highlighted the importance to patients of being treated as a ‘normal’ person with a ‘normal’ illness [16]. There was a sense that HCPs working in specialist HIV services were more understanding and accepting than general practitioners [16, 25, 27, 29], going ‘above and beyond the duties of their job’ in this respect [25]. The positive experience of sexual health services went beyond the doctor-patient relationship, with participants in one study reporting that their entire experience ‘from the receptionists to the doctors’ was friendly and welcoming [25].

### 2. Expertise of the healthcare provider

Nine studies (including 5 (56%) good quality studies) identified the expertise of the healthcare provider as being a valued aspect of care. Six quantitative studies [15, 22, 23, 32, 33, 35) demonstrated the importance to patients that the doctor they see has specialist knowledge of HIV. This included a study of patients’ satisfaction with primary care doctors, in which patients’ perceptions of their doctor’s HIV knowledge was significantly associated with satisfaction with care [35]. The importance of the healthcare provider being able to prevent illness and provide up to date HIV treatment was also evident [15, 22, 23, 32, 33]. Qualitative studies identified patients’ concerns about changes to the way their health services were provided and the increasing need to be seen by non-HIV specialists [27, 29]. Primary care physicians were perceived as having too little knowledge about HIV and lacking sufficient expertise or experience to treat HIV positive patients [22, 27, 29].

However, it was suggested that primary care physicians in rural settings should be provided with training in HIV in order to combat perceived stigma and isolation among patients in these communities [24].

### 3. Access to healthcare

Nine studies (including 4 (44%) good quality studies) found that easy access to healthcare services was important to patients. Patients valued having convenient clinic hours, being able to call the clinic, being able to make an appointment within 24 h having access to a walk-in/emergency clinic, as well as transparency (e.g. allowing patients access to their personal data and allowing patients to seek a second opinion) and reliability (e.g. doctor keeping appointments, organizing his/her replacement when not present) [13, 15, 19, 23, 32, 33]. In multivariable analysis, ease of calling the clinic and getting answers was associated with satisfaction with care, however, ease of getting to the clinic and parking were not associated with satisfaction [13]. Findings from qualitative studies revealed that patients did not want to have to wait too long to get an appointment and valued a timely response to telephone calls [20, 24]. Having enough time for discussion with nurses and doctors was also important [24]. Patients described difficulties accessing primary care, including difficulties in getting urgent appointments and insufficient consultation time, whereas specialist HIV services were perceived to be more flexible and accessible [27].

### 4. Provision of information and support

Fourteen studies (including 8 (57%) good quality studies) identified the importance to patients of information and support. It was important to patients that information was clear and easy to understand [15, 23, 32, 35]. HIV treatment information, including an explanation of treatment side effects in language that the patient could easily understand, information on how to take prescribed medication and an explanation of the advantages and disadvantages of any treatment was particularly important [15, 23, 32, 35]. Being given laboratory test results, and having them explained, were also rated as important [15, 23]. Understanding the doctor’s instructions was significantly associated with satisfaction with care [35].

Qualitative studies illustrated the importance of accessible information to facilitate understanding [17, 20, 25, 26]. This included entertaining patient’s questions and responding in in language appropriate for the individual patient, free from unfamiliar medical terms [25]. Having adequate time to discuss information about HIV as well as thoughts and feelings was also important [20]. For participants in one study, having an HIV specialist pharmacist onsite was considered important for providing up to date information about medications and treatment side effects [24].

Other types of information and support valued by patients included help with financial planning [17, 19], immigration support [20, 31] and housing advice [20, 31]. Peer support was particularly valued [16, 17, 20, 27], including informal support from a partner or friend, befriending or mentoring schemes, and support groups, including specific support groups for people facing similar issues [17, 20]. One study compared the preferences of older (age 50 years or older) and younger patients (age 20–39 years) [30]. Primary care, dental care, case management and AIDS drug programmes were rated as important by over 50% of both groups, however, older patients were more likely to value additional services such as physiotherapy, adult day care, home chore services and home delivered meals [30].

### 5. Good communication between services

Six studies (including 4 (83%) good quality studies) found that patients valued good communication between the health care professionals involved in their care. Participants in one study reported that changes to health policy had resulted in their care being fragmented between GPs, the HIV clinic and other hospital departments, with poor communication between the various services [29]. Participants in another study described the fragmentation of the healthcare system as a barrier to engagement with care [17]. To ensure continuity of care across services, participants in one study felt that it was important that their health information was shared between their HCPs within and outside the HIV clinic [24]. However, while patients in this study, especially those living with rural areas, saw the value of sharing information electronically, they expressed concerns about the security of this system in keeping their health information confidential. For some participants, this stemmed from worries about employers finding out that they were HIV positive [24].

Patients valued help linking them to different resources in the community (e.g. financial services, housing services and mental health services) [20, 24, 27]. Some patients envisaged an advocacy role for the HIV clinic in helping wider services and the public to understand issues faced by HIV positive patients [24]. This included educating employers and health insurance companies about treatments for HIV [24].

### 6. Factors relating to confidentiality and stigma

Eight studies (including 4 (50%) good quality studies) found that patients were concerned about their HIV status being kept confidential [15, 16, 23, 24, 28, 29, 31, 33]. In one study, patients were consulted on the future design of their sexual health and HIV clinic [31]. The vast majority agreed that the design of the building/environment should allow them to maintain their confidentiality, however patients differed in terms of the ways in which this should be achieved. The majority of patients did not want reception or waiting areas to be separated for HIV and sexual health, but preferred them to be divided by gender [31].

In qualitative studies, patients expressed concern about incidental disclosure of their HIV status as a result of being seen entering the clinic or being present at the clinic [21, 28]. In a study with injecting drug users, fear of disclosure of HIV status played a major role in the decision not to access care [21]. Increasing need for patients to access non-HIV medical specialties led to fears about loss of confidentiality, and this was a barrier to integration of HIV care into mainstream care [27]. In one study, patients reported concerns about the confidentiality of their HIV status in primary care [29].

In another, people from African communities expressed concerns about a specialist service for African immigrants because of the potential for disclosure of HIV status within the group [16]. HIV-related stigma was also an issue for patients when considering the introduction of new technology, such as electronic health records and futuristic smart cards that carry their health information. While patients anticipated that these would have advantages in terms of convenience and speed of data transmission, they worried about issues of confidentiality and discrimination [24].

### 7. Patient involvement in healthcare

The findings of six studies (including 2 (33%) good quality studies) indicated that it was important to patients that they were involved in decisions about their care [15, 22, 24, 28, 29, 35]. Patients who perceived they were involved in the medical encounter reported greater satisfaction with their care [35]. Involvement in healthcare included collaborating or partnering with healthcare professionals to optimize care [15, 24,25 28], having the final say in treatment decisions [22], becoming expert patients [29], and requesting copies of letters and test results in order to maintain their own medical record [29]. In order to achieve their ideal of best care, several participants in another study expressed the desire to establish a community advisory panel, which would be part of the clinical decision making process [24]. In contrast, participants in one study explained that managing one’s own health is hard to achieve in reality, therefore they wanted to rely on healthcare professionals [27]. In this study, those who had been diagnosed with HIV for longer felt more empowered and had a greater sense of knowledge and control over their condition than those more recently diagnosed.

### The relative importance of different aspects of care

While no studies examined the relative importance of all of the aspects of care identified in this review, six papers (4 (67%) rated good quality) assessed the relative importance of selected aspects of care. The most valued aspects of care identified in each of these studies are listed in Table 5. The authors of one study developed a questionnaire to assess satisfaction with a range of aspects of HIV care [13]. They then explored the relationship between each component and overall satisfaction, to gauge the relative importance of the different aspects of care. The main predictor of overall satisfaction with care was satisfaction with the HIV care provider (comprising likelihood of recommending provider, trust with provider, feelings about provider, intention to switch provider), which accounted for almost half of the variance. A card sorting exercise also found that the relationship with care providers was perceived to be the most important aspect of HIV care [22].

**Table 5:** Most valued aspects of care

Study	Measure Used	Most Valued Aspects of Care
Dang et al. (2012)	Developed a 22-item questionnaire based on validated tools, exploring perceptions of various aspects of the care provided and overall satisfaction.	The aspects of care most strongly associated with overall satisfaction were: 1) Satisfaction with the HIV provider (e.g. doctor, nurse) 2) Facility environment (e.g. noise, cleanliness) 3) Ease of calling the clinic and getting answers 4) Clinic staff (e.g. receptionist)
Davis-Michaud et al. (2004)	Participants were given 18 attributes of care on cards and asked to sort into piles according to the level of importance.	The most important factors: 1) Relationship with care providers 2) Prevention of opportunistic infections 3) Involvement in care and treatment decisions 4) Being offered ART
Hekkink et al. (2003)	QUOTE-HIV – participants rated the importance of 27 aspects of HIV care delivered by GPs, specialist doctors and nurse consultants.	Most important aspects of care: Specialists 1) Have special knowledge of HIV 2) Give information about possible side effects of drugs 3) Inform me about the pros and cons of a treatment 4) Give information about the use of my HIV medication GPs 1) Take me seriously 2) Maintain confidentiality about my HIV status 3) Take my opinion into account 4) Inform me about the pros and cons of a treatment HIV Consultant Nurse 1) Have special knowledge of HIV 2) Take me seriously 3) Give information about the use of my HIV medication 4) Maintain confidentiality about my HIV status
Hekkink et al. (2005)	QUOTE-HIV – participants rated the importance of 27 aspects of care received from HIV nurse consultant.	Aspects of care rated most important: 1) Has special knowledge about HIV 2) Takes me seriously 3) Maintains confidentiality about my HIV status 4) Can easily be reached by phone
Hope et al. (2001)	A questionnaire was developed for the study assessing the importance of a range of service attributes.	Aspects of care rated as ‘essential’ by >75% participants: 1) Treatment by specialists 2) Up-to-date treatment 3) Caring clinic 4) Individual requirements 5) Efficient outpatient service 6) Walk in service
Moore et al. (2010)	QUOTE-HIV – participants rated the importance of the various aspects of care they receive from their specialist.	Aspects of care rated most important: 1) Provides an explanation, in language that I can understand, concerning prescribed medicines 2) Tells me what the possible side effects of a medicine are 3) Provides information about how I should take the prescribed HIV medication 4) Includes me in decision making regarding the treatment that I receive

Three studies used the QUOTE-HIV questionnaire to assess important aspects of the care delivered by HIV health professionals (specialist doctors/primary care physicians/consultant nurses) in more detail [15, 23, 32]. Having specialist knowledge of HIV, taking the patient seriously and the provision of information were rated as particularly important aspects of care. Treatment by HIV specialists was rated the most important feature of aspect of care (rated essential by 89% of participants) in another questionnaire-based study [33].

## Discussion

This systematic review identified twenty-three studies that explored valued aspects of care among people living with HIV, from which seven main themes emerged. These themes may be useful in the future planning of services to meet the needs of an ageing population, including the management of multimorbidity, and may have relevance both to people living with HIV and those with other long- term conditions.

The most common theme reported in the studies related to interpersonal aspects of care. Personal qualities of health care professionals (such as being compassionate, approachable or friendly) may be difficult to quantify or modify. However, other relational aspects of care identified in this review, such as seeing the same primary or secondary care physician repeatedly, are amenable to intervention. Continuity of care has previously been identified as important to HIV positive patients attending primary care consultations [37], and is considered to be increasingly important to patients as they age and develop multimorbidity or become socially isolated [38]. Continuity could be achieved, for example, by providing patients with sufficient opportunities and choices to see the same clinician and ensuring that there is enough time in consultations for a relationship to develop [38].

It was important to patients that health care professionals involved in their care had specialist, up to date expertise in HIV. Non-HIV physicians were often perceived to have insufficient knowledge and experience of HIV and its treatment. The implications are that in order for HIV care to move beyond specialist services, there is a need for training for healthcare professionals, both in terms of up to date clinical knowledge and awareness of the particular psychosocial issues surrounding HIV. Models that have been proposed and/or implemented to address this issue include locally enhanced primary care services, in which the non-HIV care of HIV positive patients is provided by primary care physicians who receive annual training in HIV medicine and a transitional model whereby a primary care physician is available to see patients in the HIV clinic [39]. Given the increasing prevalence of age-related comorbidities in this population, the involvement of primary care physicians in the care of HIV positive patients should be reinforced and encouraged by HIV specialists [9].

Several practical considerations also emerged. People living with HIV valued being able to access care quickly and efficiently within HIV services, and this contrasted with their experiences of accessing primary care [27, 29]. Patients appeared to overlook shortcomings such as car parking and waiting times as long as they had positive experiences with their HCP [12]. A previous report identified several recommendations for improving the primary care of HIV positive patients. These included increasing appointment time, providing training for primary care physicians and practice staff to increase awareness of the concerns of HIV positive patients, and training for HIV positive patients to help understand the role of the primary care physician, identify and access primary care physicians and provide support around disclosure of HIV status [39].

Up to date, accessible information about HIV and its treatment was considered important [15, 25, 26, 35]. Patients wanted sufficient time to process and discuss information. Peer support was recognized as a valuable addition to clinical services. Participation in peer support interventions, including online support groups, has been linked to better psychological health, reduced loneliness and depression [40], improved self-care [41] and improved adherence [42]. Befriending or mentoring schemes may be a useful addition for people who do not receive informal support from a partner or friend [20]. Information and support needs may change as people age with HIV.

Fragmentation of the health care system was identified as a barrier to engagement with services [28]. Patients wanted joined up care, so that different services worked together to reach a common goal [24]. They valued the help they received from specialist HIV services in linking them to other services within the community such as financial, housing, immigration and mental health services [24]. The need for support for older people with HIV on financial, housing and mental health issues has previously been highlighted [43] and is congruent with current policy directives to better meet the needs of an ageing population by joining up health and community care [44].

HIV remains a stigmatized condition and many patients in the reviewed studies highlighted the need for their HIV status to be kept confidential. This is particularly relevant in the context of the introduction of new technologies and in the implementation of new models of care. Several reports document instances of discrimination against people with HIV in non-specialist, primary care and dental services [39, 43, 45] leading to recommendations for education for GPs and practice staff to raise awareness and tackle discrimination, and for the implementation of systems to report incidences of discrimination in healthcare [39].

### Limitations

The search was challenging because the topic was broad. We are confident that we included all the relevant studies, having screened over 5500 titles and abstracts. There has been very little quantitative research in this area. The reviewed studies used diverse methods and assessed different aspects of care, therefore it was not possible to compare findings across studies, or between different populations. Our inclusion criteria specified that the primary aim of the paper or element of the results was to explore which aspects of health care were valued by people living with HIV. The rationale for this was to ensure that studies included in the review were relevant to the research question, however, it is possible that some of the studies that were excluded because they did not meet this criterion included some information about valued aspects of care. The evidence for some of the themes (access to healthcare and patient involvement in healthcare) was weak, due to the low quality of the studies contributing to these themes.

The extent to which the findings of this study can be used to inform the design of a new approach to care is limited in the following ways: 1) many of the valued aspects of care that were identified in this review related to personal qualities of the healthcare professional (e.g. taking the patient seriously) for which there is not an obvious solution; 2) it was not always clear what aspects of care were driving satisfaction; 3) there was some inconsistency in the findings, for example while some participants wanted their HIV clinic to be separate from sexual health services, for others, segregation by gender was preferable. There are also limitations in terms of the generalizability of the findings. The mean age of participants in all studies reviewed was less than 50 years, although one study [29] employed quota sampling to ensure representation of people aged >50 years, and another compared the needs and preferences of older and younger groups [30]. The findings of the review indicate that there has been little relevant research to identify valued aspects of care among older adults, who are at greater risk of multimorbidity [6]. Furthermore, we only included studies conducted in high income countries with a history of good access to ART. The results may therefore not be generalizable to people living with HIV in countries in which there have been more barriers to ART access, such as those in sub-Saharan Africa, where issues of ageing and multimorbidity are also relevant [46]. Further research is required to determine whether preferences for the delivery of care differ between different demographic groups, such as between older and younger patients.

## Conclusions

The findings of this systematic review highlight aspects of healthcare that are valued by people living with HIV and may facilitate the systematic development of evidence-based interventions to improve services and ultimately enhance patient outcomes and experience in the setting of a changing epidemic with an ageing population. Quantitative research to examine the relative importance to patients of these valued aspects of care, and to identify any differences across demographic groups is currently being conducted by this research group. The intention is to ensure that the views and preferences of people with HIV can inform the development of new services, thereby maintaining high levels of patient satisfaction and engagement with their care.

## Abbreviations

ART: Antiretroviral therapy
GP: General Practitioner
GUM: Genitourinary Medicine
HIV: Human Immunodeficiency Virus
HCP: Health Care Professional
MSM: Men who have Sex with Men
NHS: National Health Service
NIHR: National Institute for Health Research
